# An Important Meeting

**Published:** 1898-02

**Authors:** 


### AN IMPORTANT MEETING.

THE ninth International Congress of Hygiene and Demography will be held in Madrid, Spain, April 10 to 17, 1898. On another page we give a synopsis of the subjects connected with veterinary science on which papers are solicited. Several of these topics are of much importance to us at this particular time, and we trust that some of our American colleagues will forward papers bearing upon the same. The importance of these gatherings can hardly be overestimated, as they give national importance to our work and impetus to reform movements that are of vital importance to our welfare as a profession. We wish the Congress the greatest possible success, and note with pleasure the progressive spirit of our Spanish colleagues in promoting the undertaking.

